# Visual circuits in arthropod brains

**DOI:** 10.1007/s00359-020-01407-9

**Published:** 2020-02-08

**Authors:** U. Homberg

**Affiliations:** Fachbereich Biologie, Philipps-Universität Marburg, 35032 Marburg, Germany

Many arthropods, notably spiders, decapod crustaceans, and insects have excellent visual systems that allow them to evaluate self-movement and environmental features in many different ways. Objects can be discriminated based on luminance contrast, color contrast, and polarization contrast, and moving objects can be traced in front of moving backgrounds. Thus, various qualities are used for the identification and pursuit of conspecifics and prey, for the escape from predators, for monitoring self-movement, for maintaining orientation in space, and on a more basic level, for the entrainment of internal circadian clocks. The neuronal machinery evaluating the inputs from the eyes can be enormous and, in many species, encompasses more than three quarters of all brain neurons (Strausfeld 1976). In insects and crustaceans, primary visual processing takes place in a sequence of optic neuropils, termed lamina, medulla, and lobula complex that are retinotopically organized into layers and columns. Further processing, usually not organized retinotopically and including integration with other sensory modalities and motor control networks, involves large additional areas in the central brain and ventral nerve cord.

Despite this complexity in visual signal processing, the repetitive nature of retinotopic modules in the optic lobes and the existence of individually identifiable neurons have favored arthropod, and in particular, insect visual systems as models for the analysis of visual functions at the level of single-identified neurons and neuronal networks. This special issue highlights particular areas of active research aimed at uncovering neuronal networks underlying visual functions such as motion vision, depth perception, color vision, polarization vision, and circadian clock entrainment. Owing to unique molecular genetic techniques and ongoing research toward establishing the complete connectome of the brain (Scheffer and Meinertzhagen 2019), research in these areas increasingly focusses on the fruit fly *Drosophila melanogaster.* This is also reflected in this special issue. Seven out of 13 articles focus on research in *Drosophila*. In addition, comparative studies in hoverfly, praying mantis, butterfly, grasshopper, and stomatopods elucidate neural mechanisms underlying particular visual adaptations such as target pursuit, predator approach, prey capture, and mechanisms of color vision.

Neural networks underlying *motion sensitivity* are particularly well understood. Based on behavioral data in a beetle, Reichert and Hassenstein (1956) proposed a model for an elementary motion detection circuit in the insect brain. Research over the last 60 years largely supports this model and has now identified virtually every neuronal cell type involved in the motion vision circuitry in the fly brain. Haag and Borst (2020) summarize the current state of knowledge, provide a step-by-step guidance through the motion vision circuit, and address future directions of research, partly aimed at uncovering synaptic transmission mechanisms within the network. Research in various insect species, notably flies, bees, and locusts has shown that motion vision circuits in the optic lobe are modulated in various ways and at different levels of processing by nutritional status, current behavior, environmental changes, or novel sensory cues. Pharmacological experiments show that centrifugal neurons releasing biogenic amines, in particular serotonin and octopamine, mediate parts of these effects. Cheng and Frye (2020) review the state of knowledge on modulation of visual circuits in insects, address the limitations of current pharmacological evidence, and point out the benefits in future experiments of manipulating selected modulatory neurons in *Drosophila* using optogenetic tools.

Feedback from large-field motion-sensitive neurons of the lobula plate on motor pathways is required in flies to control *flight stability* during flight maneuvers. Traditional electrophysiological studies have identified nearly 60 different types of these neurons in blowflies, but only a fraction of them in *Drosophila*. The paper by Wei et al. (2020) used systematic Gal4 driver screens to identify novel types of lobula tangential neurons in *Drosophila* that are morphologically similar to those identified earlier in the blowfly. Based on these data, the functional role of these neurons in *Drosophila* in motion computation and signaling can now be addressed. Insect flight maneuvers are generally under strong visual control. Nicholas et al. (2020) have analyzed visual motion sensitivity in descending neurons of the hoverfly, *Eristalis tenax,* that project from the brain to the ventral ganglia and likely take part in flight control. Based on their receptive field properties, neurons could be classified as being selective to small moving targets, looming sensitive, and optic flow sensitive, suggesting that these classes of neurons are involved in different visually controlled behavioral tasks.

In most arthropods, *perception of distance* of objects relies on angular motion cues. During ego motion, the projections of nearby objects move faster across the retina than those of distant objects. Praying mantises, in addition, use stereoscopic cues based on differences in viewing angle of the right and left eye to judge the distance of prey-like targets. The optic lobe, especially the lobula complex, of these insects shows a number of highly specific organizational features not found in other insects. Rosner et al. (2020) used sophisticated 3D virtual stimulation devices to analyze the binocular tuning of projection neurons of the medulla and lobula complex of these insects that are likely to participate in the stereoscopic network.

In contrast to a wealth of studies on motion vision, the neural networks underlying *color vision* have been studied less well and principles of neural processing and interactions are just beginning to be elucidated. The section on color and polarization vision starts with a comprehensive review of current knowledge on neural mechanisms underlying color vision in *Drosophila* and other insects (Schnaitmann et al. 2020). The authors review the role of color vision in insects and evidence for the emergence of color opponent pathways in the lamina and medulla. They point out promising research strategies for the analysis of the neuronal basis of phenomena such as spatial and temporal color contrast and color constancy.

The color vision system of the swallowtail butterfly *Papilio xuthus* is tetrachromatic. Therefore, it is considerably more sophisticated than the trichromatic color vision system in honeybees and other insects. Following the identification of photoreceptor types and their distribution in the eye, Chen et al. (2020) investigated early mechanisms of chromatic processing at the level of the lamina of the butterfly. Using electrophysiological techniques, the authors show spectral opponency in photoreceptors as well as in a subclass of lamina monopolar neurons. Together with three types of non-opponent lamina monopolar neurons, these elements likely serve as input for further color processing in the medulla. In contrast to first groundbreaking data on color processing mechanisms in the optic lobe, the chromatic properties of neurons in the central brain have only been studied in a few insect species, notably bees, but are largely unexplored in *Drosophila*. Yonekura et al. (2020) have, therefore, used in vivo whole-cell patch-clamp technology in a pioneering study to record and characterize the chromatic properties of interneurons in the central brain of *Drosophila*. Most neurons, usually projection neurons from the lobula complex targeting various regions of the central brain, were sensitive to broad-band chromatic stimuli either in the short or middle wavelength range, suggesting that they receive input from different types of photoreceptor. The significance of the apparent lack of color opponent responses is discussed.

In many insect species, a small dorsal part of the eye, lamina, and medulla is specialized to signal the polarization pattern of the sky for navigation purposes. Sancer et al. (2020) show in *Drosophila* that the dorsal rim of the medulla, which is a region specialized for *polarization vision*, differs in several aspects from the rest of the medulla. Certain cell types are completely missing, others show differences in structure or connectivity, and octopaminergic modulatory input is conspicuously absent. The data provide the basis for a functional analysis of early processing of polarization signals and suggest that more than one pathway connects the dorsal rim area to the central brain.

Stomatopod crustaceans have one of the most complex visual systems among arthropods. Particularly prominent is an equatorial midband of six rows of ommatidia that serve specific roles in color and polarization vision. Certain species only possess two rows of midband ommatidia. Lin et al. (2020) have investigated structural differences between the six-row and two-row species in the optic lobe. They show that the two-row species lack particular features both in medulla and lobula neuropils and suggest that the two-row species have secondarily lost four color-sensitive rows of midband ommatidia owing to their habitats in deep waters, muddy environments and a nocturnal activity period.

In addition to their role in color, polarization, and motion vision, photoreceptors provide an essential non-image forming function in light entrainment of the *circadian clock*. Helfrich-Förster (2020) shows that, in addition to the expression of the blue-light sensitive photoreceptor cryptochrome in *Drosophila* clock neurons, the role of the compound eyes, ocelli, and various extraretinal photoreceptors in light entrainment of the clock is highly species specific in different insect taxa. Recent data from *Drosophila* provide evidence for different roles of these photoreceptors depending on daylength and light levels and are beginning to show how circadian clock neurons integrate the different photic inputs for environmentally adaptive activity rhythms.

A comprehensive understanding of visual circuits not only requires identifying the contributing neuronal cell types, but also an understanding of synaptic mechanisms, neurotransmitters and the cell type specific *expression of ion channels*. Two contributions toward these directions are provided by Gür et al. (2020) and Wang et al. (2020). Gür et al. (2020) analyzed the basis of membrane properties of lamina monopolar neurons at the first stage of processing in the motion vision system of *Drosophila*. The authors show that two types of voltage-gated potassium channel are expressed in a particular subtype of lamina monopolar neurons (L2) mediating the lights-off pathway. Knockdown experiments showed that one of these channels is essential in shaping the fast temporal dynamics that are specific for L2 monopolar neurons. The study thus reveals essential data on the basis of signaling dynamics at the first processing stage of motion vision circuitry.

As part of their analysis of visually triggered escape pathways, Wang et al. (2020) studied the distribution of voltage-gated sodium channels (NaV) in the brain of locust and fruitfly. The authors identified a *Drosophila* ortholog of a NaV channel sequence in the transcriptome of the locust and used immunolabeling to analyze the distribution of NaV in the locust and fly brain. The data show that NaV channels are highly conserved among insects and open the way for future analysis of the subcellular localization of the NaV channel in the locust visual escape pathway.

Taken together, the articles in this special issue not only highlight exciting novel discoveries in arthropod visual neuroscience, but also address a wealth of open questions that are likely to inspire future investigations. I am extremely grateful to all authors who contributed stimulating articles to this special issue, thereby illustrating the richness of topics in arthropod visual circuit analysis. I also thank the reviewers for their constructive criticisms and helpful suggestions that substantially improved the clarity and overall quality of all manuscripts.
